# Peptide-Functionalized Nanomedicine: Advancements in Drug Delivery, Diagnostics, and Biomedical Applications

**DOI:** 10.3390/molecules30071572

**Published:** 2025-03-31

**Authors:** Hossein Omidian, Luigi X. Cubeddu, Renae L. Wilson

**Affiliations:** Barry and Judy Silverman College of Pharmacy, Nova Southeastern University, Fort Lauderdale, FL 33328, USA; rw1273@mynsu.nova.edu

**Keywords:** peptide-functionalized carriers, targeted drug delivery, molecular imaging and biosensing, regenerative medicine, nanotechnology in therapeutics

## Abstract

Peptide-functionalized nanomedicine has emerged as a transformative approach in precision therapeutics and diagnostics, leveraging the specificity of peptides to enhance the performance of nanocarriers, including gold nanoparticles, polymeric nanoparticles, liposomes, mesoporous silica nanoparticles, and quantum dots. These systems enable targeted drug delivery, molecular imaging, biosensing, and regenerative medicine, offering unparalleled advantages in bioavailability, cellular uptake, and therapeutic selectivity. This review provides a comprehensive analysis of peptide-functionalization strategies, nanocarrier design, and their applications across oncology, neurodegenerative disorders, inflammatory diseases, infectious diseases, and tissue engineering. We further discuss the critical role of physicochemical characterization, in vitro and in vivo validation, and regulatory considerations in translating these technologies into clinical practice. Despite the rapid progress in peptide-functionalized platforms, challenges related to stability, immune response, off-target effects, and large-scale reproducibility remain key obstacles to their widespread adoption. Addressing these through advanced peptide engineering, optimized synthesis methodologies, and regulatory harmonization will be essential for their clinical integration. By bridging fundamental research with translational advancements, this review provides an interdisciplinary roadmap for the next generation of peptide-functionalized nanomedicines poised to revolutionize targeted therapy and diagnostics.

## 1. Introduction

Peptide-functionalized (PF) products combine the high specificity and bioactivity of peptides with the versatility of nanocarriers, biomaterials, and drug delivery systems. These hybrid platforms have garnered significant attention in targeted drug delivery, molecular imaging, biosensing, regenerative medicine, and antimicrobial applications, offering enhanced therapeutic efficacy, bioavailability, and disease detection sensitivity [1,2,3,4]. By leveraging tumor-homing, blood–brain barrier-penetrating, antimicrobial, and bioadhesive peptides, researchers have engineered functionalized carriers that achieve site-specific interactions, improved cellular uptake, and controlled drug release [5,6,7,8,9,10].

The biomedical potential of PF systems stems from their ability to target specific cellular receptors, regulate biological interactions, and enhance therapeutic selectivity. In cancer therapy, PF nanosystems have been widely employed to improve tumor penetration, co-deliver drugs, and overcome chemoresistance, particularly for aggressive and drug-resistant malignancies [6,9,11,12,13]. Additionally, PF platforms have transformed gene therapy by improving siRNA, miRNA, and CRISPR-Cas9 delivery, which remain hindered by poor cellular uptake and enzymatic degradation [14,15,16,17]. Beyond oncology, neurological applications have demonstrated the potential of PF liposomes, nanoparticles, and extracellular vesicles in enabling drug penetration across the blood–brain barrier (BBB) and targeting neurodegenerative disease hallmarks [18,19,20,21,22].

Beyond therapeutic applications, PF biosensors, imaging agents, and implant coatings have paved the way for early disease detection, precision diagnostics, and regenerative medicine. In cancer imaging, PF magnetic resonance imaging (MRI) contrast agents, gold nanostructures, and quantum dots have enhanced tumor biomarker detection and real-time disease monitoring [4,23,24,25,26,27,28]. Regenerative medicine has similarly benefited from PF scaffolds, hydrogels, and titanium coatings, which improve osseointegration, wound healing, and stem cell expansion while reducing implant-associated infections [10,29,30,31,32,33,34,35]. Additionally, antimicrobial and antiviral PF surfaces and nanoparticles have demonstrated promising applications against multi-drug-resistant bacteria and emerging viral threats such as SARS-CoV-2 [10,34,36,37,38,39].

Despite significant advancements, several challenges remain regarding their stability, immune response modulation, long-term toxicity, scalability, and clinical validation. Peptides are often subject to enzymatic degradation and systemic clearance, requiring chemical modifications such as PEGylation, cyclization, or non-natural amino acid incorporation to enhance their half-life and therapeutic retention [9,10,40,41,42,43]. Additionally, manufacturing hurdles and batch-to-batch variability limit their large-scale production, requiring the standardization of conjugation techniques, purification strategies, and regulatory compliance [10,17,44,45]. This review provides a comprehensive and interdisciplinary analysis of PF products, detailing their design principles, material selection, biomedical applications, and translational challenges while addressing the scientific and engineering innovations necessary to overcome current limitations.

## 2. Peptide Functionalization of Drug Delivery Carriers

Peptides have been widely utilized in drug delivery systems due to their specificity, biocompatibility, and ability to enhance targeting and cellular uptake. The functionalization of drug carriers with peptides has been shown to improve drug bioavailability, reduce systemic toxicity, and facilitate precise therapeutic delivery. A variety of peptides have been investigated for this purpose, targeting integrins, tumors, specific receptors, and cellular entry pathways.

### 2.1. Integrin-Targeting Peptides

Integrin-targeting peptides facilitate selective binding to integrins, particularly αvβ3 and αvβ5, which are overexpressed in tumor vasculature. The cyclic arginylglycylaspartic acid peptide (cRGD) [1,2,11,13,31,46,47,48] has been extensively used to functionalize nanoparticles, enabling targeted tumor delivery through high-affinity binding to αvβ3 integrins. Its cyclic conformation enhances its stability, specificity, and receptor-binding affinity, making it superior to its linear counterpart for tumor-targeting applications. Another integrin-targeting peptide, the fibronectin-derived RGD peptide [46], has been investigated for vascular graft integration and endothelial cell retention, leveraging its natural affinity for integrins. While not primarily used for drug delivery, its role in promoting endothelialization and biomaterial integration highlights its relevance in targeted vascular applications. Additionally, thiol-functionalized RGD peptides [48] have been employed for surface functionalization, facilitating cell adhesion and detachment studies on biomaterials. The thiol (-SH) group allows for strong conjugation with gold and other nanoparticle surfaces, improving stability and enhancing biointerface interactions.

### 2.2. Tumor-Homing and Penetrating Peptides

Tumor-homing and -penetrating peptides enable the selective accumulation of drug carriers within tumor tissues, enhancing drug efficacy and reducing off-target toxicity. The F3 peptide [9,49,50] binds to nucleolin, a protein overexpressed on tumor endothelial and cancer cells, facilitating nanoparticle retention within tumors. This peptide has been incorporated into various nanoparticle formulations to enhance targeted cancer therapy and tumor imaging, particularly in breast cancer applications. The CooP tumor-homing peptide [51] exhibits selective affinity for brain tumors, aiding in drug penetration across the blood–brain barrier (BBB). Its integration into nanoparticle-based drug carriers has shown promise for targeted glioblastoma therapy. The internalizing RGD (iRGD) peptide [16] is a dual-function tumor-penetrating peptide, interacting with αvβ3 integrins and neuropilin-1 (NRP-1). This C-end rule (CendR) mechanism enhances deep tissue infiltration, making iRGD a valuable tool for improving drug penetration in dense tumor environments. Studies have demonstrated its potential in siRNA-based lung cancer therapy, where it facilitates efficient tumor cell internalization and distribution.

### 2.3. Cell-Penetrating Peptides (CPPs)

Cell-penetrating peptides (CPPs) enhance intracellular drug delivery by facilitating membrane translocation, enabling the efficient transport of therapeutic agents across biological barriers. The TAT peptide, derived from the HIV-1 transactivator of transcription (TAT) protein, is one of the most extensively studied CPPs [17,21,52,53,54,55,56,57]. It enables receptor-independent uptake through mechanisms such as macropinocytosis, allowing nanoparticles to deliver drugs intracellularly. Variants like TAT-NLS and TAT-AT7 have been developed for genome editing and glioma-targeted gene delivery, respectively. Another well-characterized CPP, the penetratin peptide, originates from the Antennapedia homeodomain and has been used to improve drug uptake into brain tissues and across the BBB [57,58]. This property has been leveraged in siRNA delivery for inflammatory diseases and neurodegenerative disorder treatments, including Alzheimer’s disease therapy. The Selective Cell-Penetrating Peptide (SCPP) [59] has been engineered to enhance selective internalization into cancer cells, reducing the off-target effects associated with conventional CPPs. Its role in targeted lung cancer therapy using methotrexate-loaded polymersomes demonstrates its potential in improving drug specificity. Additionally, a fusion peptide combining RGD with octa-arginine (R(8)) [13] been developed, integrating integrin-targeting and CPP properties. This dual-function peptide enhances tumor-selective intracellular delivery, offering a synergistic approach to targeted drug transport.

### 2.4. Receptor-Targeting Peptides

Receptor-targeting peptides enable specific drug delivery through receptor-mediated endocytosis, improving targeting precision and reducing off-target effects. The CD44-binding peptide [3] targets CD44 receptors, which are overexpressed in various tumors, facilitating active nanoparticle uptake in cancer cells. This strategy has been employed in oil core-based nanocapsule formulations to enhance cancer cell targeting. Neuropilin-1-targeting peptides (CPL-K, CPL-F) [43] interact with NRP-1, a receptor involved in tumor angiogenesis, to improve drug accumulation in tumor tissues. These peptides have been incorporated into nanocarriers for anticancer peptide delivery. The transferrin-binding peptide (TBP) [60] binds to the transferrin receptor (TfR), which mediates receptor-mediated endocytosis, making it a valuable tool for brain-targeted drug delivery. Its application in targeted doxorubicin delivery for colorectal cancer highlights its potential beyond neurological applications. The galectin-1-targeting peptide (P7) [24] selectively binds to galectin-1-expressing tumors, a protein linked to immune evasion and tumor progression. This peptide has been used in the molecular imaging of thyroid cancer, leveraging ultra-small superparamagnetic iron oxide nanoparticles (USPIOs) for enhanced detection. The HER2-targeting peptide [61] is specifically designed for HER2-positive breast cancer. Rather than direct therapy, it plays a crucial role in HER2 status detection in circulating tumor cells (CTCs), aiding in breast cancer diagnosis and treatment efficacy prediction. Finally, glypican-3-binding peptides (GPC3) [26,40,62] have been identified as selective targeting agents for hepatocellular carcinoma (HCC). These peptides have been incorporated into various nanoplatforms, including human serum albumin (HSA) particles and gold nanoparticles, enabling targeted imaging and therapy for liver and ovarian cancers.

### 2.5. Antimicrobial Peptides (AMPs)

**Figure 1:**
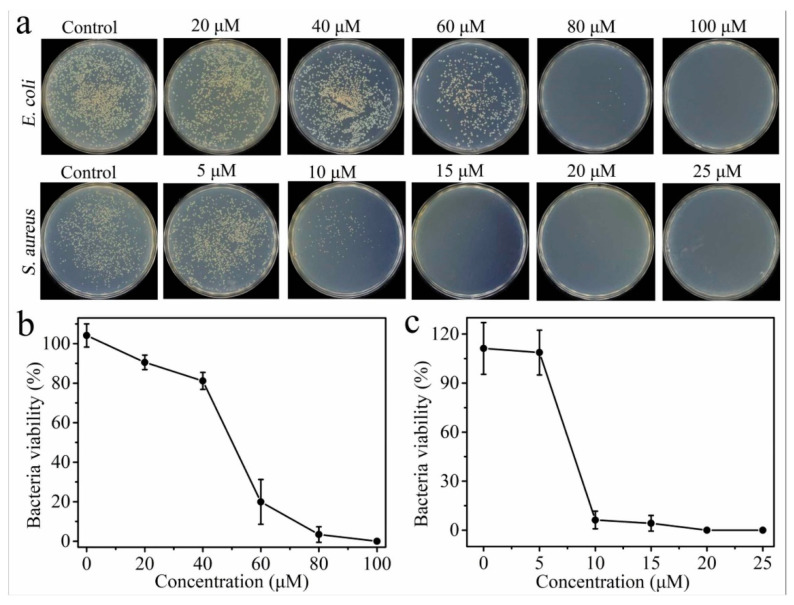
(**a**) Photographic images of the colonies of *E. coli* and *S. aureus* treated with different concentrations of Cu-GGH-AMP in Tris buffer at pH 7.5. (**b**) Viabilities of *E. coli* and (**c**) *S. aureus* calculated from (**b**) [38].

Antimicrobial peptides (AMPs) are potent membrane-disrupting agents that have been widely utilized to functionalize drug carriers to enhance antibacterial efficacy, particularly against multidrug-resistant (MDR) bacteria [10,29,34,39,63]. Various AMPs have been incorporated into nanoparticle systems to improve targeted antimicrobial therapy. For instance, the Cu-GGH-AMP (Figure 1) [38] leverages copper coordination to enhance antimicrobial activity, facilitating DNA cleavage-based bacterial disinfection and promoting wound healing. The HHC36 peptide [29], a broad-spectrum AMP, has been integrated into mesoporous silica nanoparticles (MSNs) on titanium implants, allowing for controlled antimicrobial release and improved osseointegration, reducing the risk of implant-associated infections. Similarly, SAAP-148 [34] has been employed in supramolecular coatings on titanium implants to prevent biomaterial-associated infections, demonstrating strong bactericidal activity against MDR bacteria. Another approach involves GLFVDK-Cy7, an AMP conjugated to gold nanostars (AuNS) [39], which enables photothermal therapy for Staphylococcus aureus infections, using light activation to induce bacterial membrane disruption. In pulmonary infection treatment, CGSPGWVRC and Indolicidin peptides [63] have been incorporated into chitosan-based microcapsules, facilitating the dual active targeted treatment of lung infections. Indolicidin, known for its broad-spectrum antibacterial and antifungal activity, enhances antimicrobial efficacy in respiratory applications. These receptor-functionalized antimicrobial peptides demonstrate great potential in infection control, particularly in surgical site infections (SSIs), implant-related infections, and pulmonary infections.

### 2.6. Peptides for siRNA and Gene Delivery

Peptides play a crucial role in nucleic acid delivery by enhancing the stability, uptake, and intracellular trafficking of siRNA and DNA-based therapeutics. These peptide-functionalized systems improve gene-silencing efficiency and targeted gene regulation, making them valuable in cancer therapy and genetic disorders. A peptide-functionalized siRNA system [15] has been developed to enhance siRNA delivery efficiency, particularly by improving cellular uptake and facilitating endosomal escape. This approach has been employed in multivalent gold nanorods, leading to enhanced gene silencing and metastasis inhibition in breast cancer, demonstrating its potential in RNA-based therapeutics. Peptide Nucleic Acids (PNAs) [64], which are synthetic DNA analogs with a peptide backbone, exhibit high binding affinity and resistance to enzymatic degradation, making them effective for gene therapy applications. PNAs have been functionalized onto adenoviral vectors to target G-Quadruplex structures in the Bcl-2 oncogene, a key regulator of apoptosis and cancer progression, supporting their application in anticancer gene therapy.

### 2.7. Organ-Specific Targeting Peptides

Organ-specific targeting peptides enable precise drug delivery to specific tissues, minimizing off-target effects and systemic toxicity. These peptides have been incorporated into nanoparticle-based drug carriers to improve therapeutic precision in liver cancer, obesity, and arthritis treatments. The liver cancer-targeting peptide [65] facilitates drug accumulation in hepatocellular carcinoma (HCC) cells by recognizing HCC-specific surface markers, thereby improving treatment efficacy. This strategy has been employed in gold nanoshell-based photothermal therapy, enabling selective tumor ablation while sparing healthy liver tissue. The adipose homing peptide (AHP) [66] has been developed to target adipose tissues, providing a novel approach for metabolic disorder treatment and obesity imaging. Quantum dots functionalized with AHP have been explored for the non-invasive imaging of adipose tissues, with potential applications in anti-obesity therapies. Similarly, the joint-homing peptide (ART-1) [33] was designed to direct drug carriers to joint tissues, making it a promising candidate for arthritis therapy. Liposome formulations incorporating ART-1 have demonstrated effective subcutaneous drug delivery, improving drug retention and therapeutic outcomes in joint-related diseases.

### 2.8. Functional Peptides for Drug Carrier Enhancement

In addition to their targeting and delivery functions, certain peptides improve drug carrier stability, bioavailability, and biomaterial interactions. These functional peptides contribute to enhanced drug release profiles, biocompatibility, and therapeutic efficiency. Titanium-binding peptides (TiBP1, TiBP2) [35] have been utilized for biomaterial surface modification, particularly in titanium-based medical implants. By promoting osteointegration and bioactivity, these peptides improve the adhesion of bone-forming cells, enhancing implant stability and long-term functionality. The collagen peptide [5] has been incorporated into scaffolds, hydrogels, and nanoparticle systems to improve its biocompatibility and mechanical integrity. In drug delivery applications, collagen-functionalized chitosan nanoparticles have demonstrated enhanced therapeutic efficacy in cancer treatment by improving drug retention and controlled release. The selenol-modified uPA-specific peptide [67] enhances enzymatic targeting in protease-responsive drug delivery systems, enabling precise activation in tumor environments. Its integration into mesoporous silica nanoparticles has shown promise in resveratrol delivery for triple-negative breast cancer, where selective activation by the urokinase-type plasminogen activator (uPA) ensures localized drug release. To address the low bioavailability of hydrophobic drugs, the curcumin–lipid ligand [68] was designed to enhance solubility and cellular uptake. This formulation, when incorporated into nanoliposomes, has demonstrated neuroprotective potential by inhibiting amyloid-beta aggregation, a key factor in Alzheimer’s disease progression.

Table 1 explores the diverse applications of bioactive peptides in drug delivery, therapy, and biomedical engineering. AMPs enhance wound healing and combat resistant bacteria, while CPPs facilitate targeted drug delivery, improving cancer and neurological treatments. Functional peptides optimize drug carrier systems for cancer therapy and immunotherapy. Integrin-targeting peptides aid in tumor targeting, vascular grafts, and tissue engineering. Lastly, organ-specific peptides enable precision drug delivery for arthritis, liver cancer, and obesity-related treatments. The table highlights a trend of nanotechnology-driven peptide functionalization, optimizing therapeutic efficiency across various medical fields.

## 3. Peptide-Functionalized Drug Delivery Carriers

The functionalization of various drug delivery carriers with peptides has been extensively studied to improve targeting, enhance therapeutic efficacy, and increase cellular uptake. A wide range of carriers, including nanoparticles, liposomes, hydrogels, and polymeric systems, have been explored for peptide functionalization. The choice of carrier depends on its physicochemical properties, ability to encapsulate therapeutic agents, and efficiency in delivering drugs to specific tissues or cells.

### 3.1. Gold Nanoparticles and Nanostructures

**Figure 2:**
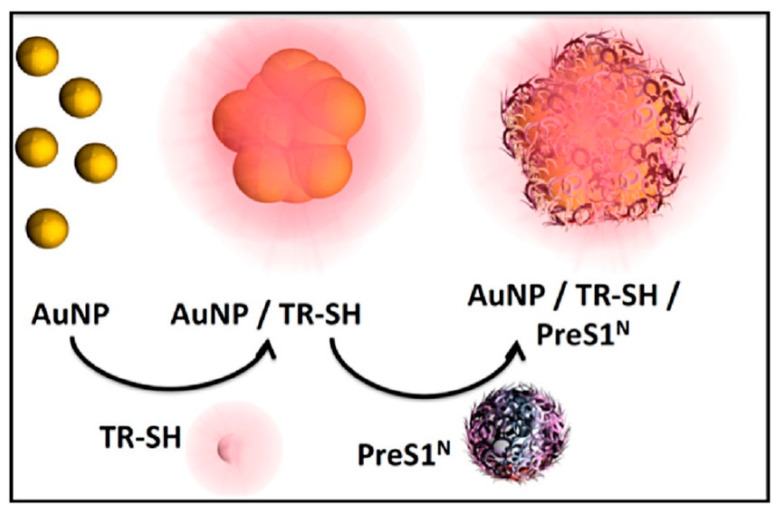
Schematic representation of the preparation of AuNP@PreS1^N^ nanostructures and other targeted nanostructures, including AuNP@mPEG. The illustration depicts AuNP@PreS1^N^ after laser ablation synthesis, showing naked nanoparticle aggregation, functionalization with the SERRS reporter TR-SH, and conjugation with the targeting ligand PreS1^N^ [23].

AuNPs are widely utilized as drug carriers due to their biocompatibility, ease of functionalization, and unique optical properties. PF-AuNPs have been extensively explored for both therapeutic and diagnostic applications, including cancer therapy, neurodegenerative disease treatment, and antimicrobial therapy. One of the most widely studied PF-AuNPs involves cRGD peptides, which have a high affinity for αvβ3 integrins, a receptor overexpressed in tumor vasculature. This property has enabled cRGD-functionalized AuNPs to be applied in targeted cancer therapy and imaging, particularly in colorectal cancer [1,2]. In neurodegenerative disease applications, cysteine–Abeta peptide-functionalized AuNPs have been developed for the early-stage detection and inhibition of amyloid-beta (Aβ) aggregation, a key pathological feature of Alzheimer’s disease [19]. Similarly, AuNPs functionalized with prohibitin (PHB)-targeting peptides have been explored for selective apoptosis induction, leveraging PHB’s role in mitochondrial function and cancer cell survival [82]. Beyond AuNPs, gold nanostructures such as gold nanorods (AuNRs) and gold nanocages (AuNCs) offer additional advantages in photothermal therapy and immune modulation. AuNCs functionalized with A54 peptide have been studied for targeted photothermal chemotherapy in liver cancer, as the A54 peptide binds specifically to hepatocellular carcinoma cells, enhancing tumor-selective drug delivery [83]. Similarly, AuNPs functionalized with PHB-targeting peptides have been explored for selective apoptosis induction, leveraging PHB’s role in mitochondrial function and cancer cell survival [82]. Figure 2 illustrates the functionalization process of AuNPs with thiolated rhodamine (TR-SH) and the PreS1^N^ peptide, highlighting the stepwise conjugation method used to enhance targeting capabilities. Beyond AuNPs, gold nanostructures such as AuNRs and AuNCs offer additional advantages in photothermal therapy and immune modulation [23]. AuNRs functionalized with RGD and GLF tripeptides have also been investigated for their impact on immune response and liver injury, showing potential in modulating inflammatory pathways in hepatitis [76]. In infectious disease treatment, gold nanostars (AuNS) functionalized with the antimicrobial peptide (GLFVDK-Cy7) have been designed for photothermal therapy against Staphylococcus aureus infections. This approach enables light-activated bacterial eradication, offering an innovative solution for treating multidrug-resistant bacterial infections [39].

### 3.2. Polymeric Nanoparticles

Polymeric nanoparticles (PNPs) have been extensively studied as drug delivery vehicles due to their stability, tunable release properties, and capacity to encapsulate both hydrophilic and hydrophobic drugs. These nanoparticles can be functionalized with peptides to improve their targeting specificity and therapeutic efficacy for various diseases. PEG-PLA nanoparticles (PP-NP) functionalized with the tumor-homing F3 peptide have been employed for chemo-photodynamic combination therapy in drug-resistant cancers, utilizing F3’s affinity for tumor endothelial and cancer cells to enhance drug accumulation at tumor sites [9]. Another peptide-functionalized polymeric system includes polyester-aminic nanoparticles modified with the iNGRt peptide, which have been investigated for targeted breast cancer therapy using docetaxel, leveraging the peptide’s tumor-penetrating properties [84]. Chitosan-based nanoparticles have demonstrated potential in brain-targeted drug delivery and cancer therapy. Chitosan nanoparticles (CS/TPP) conjugated with the TG peptide have been developed for resveratrol delivery to combat obesity-related Alzheimer’s disease, allowing for the brain-specific accumulation of neuroprotective agents [22]. Additionally, chitosan/hyaluronic acid nanogels functionalized with endothelin-1 and bradykinin receptor antagonist peptides (BQ-123 and R-954) have been explored for osteoarthritis treatment, leveraging these peptides’ ability to modulate joint inflammation and pain signaling [85]. Polymersomes, a class of self-assembling polymeric vesicles, have also been functionalized with peptides to improve targeted drug delivery. SCPPs have been used in polymersomes for targeted delivery of methotrexate disodium to lung cancer, improving cellular uptake and drug retention in lung tumor cells [59]. Similarly, TBP have been incorporated into polymersomes for the targeted delivery of doxorubicin to colorectal cancer, utilizing the transferrin receptor’s role in receptor-mediated endocytosis to facilitate drug transport across cellular barriers [60].

### 3.3. Liposomes

Liposomes, composed of lipid bilayers, have been widely functionalized with peptides to improve drug targeting, bioavailability, and cellular uptake. These peptide-functionalized liposomal systems have been applied in neurological disorders, oncology, arthritis, and pulmonary drug delivery. In neurodegenerative disease therapy, liposomes conjugated with a modified ApoE-derived peptide were developed to target Aβ aggregates, facilitating drug delivery for Alzheimer’s disease treatment [18]. Similarly, in joint disease therapy, liposomes functionalized with the ART-1 were studied, focusing on subcutaneous drug delivery in arthritis, improving drug accumulation and therapeutic effectiveness in inflamed joint tissues [33]. In oncology applications, Pep-1 peptide-modified liposomes were investigated for targeted glioma therapy using cilengitide, an integrin-targeting anticancer agent [86]. Additionally, Pep-1 and folic acid (FA) peptide-modified liposomes were employed for enhanced bladder cancer therapy, optimizing the delivery of BCG cell wall skeletons, an immunotherapeutic agent used in bladder cancer treatment [87]. For lung-targeted drug delivery, T7 peptide-functionalized liposomes were explored for pulmonary drug delivery in lung cancer therapy, leveraging T7’s affinity for transferrin receptors to enhance lung epithelial cell uptake [88]. Lastly, tumor-penetrating peptide (tLyP-1)-functionalized liposomes were developed to improve drug delivery to invasive cancer cells, enhancing drug penetration within the tumor microenvironment [69].

### 3.4. Mesoporous Silica Nanoparticles (MSNs)

Mesoporous silica nanoparticles (MSNs) serve as versatile drug delivery platforms due to their high surface area, tunable pore size, and capacity to encapsulate various therapeutic agents. These nanoparticles can be functionalized with peptides to enable targeted delivery, controlled drug release, and enhanced bioactivity in different disease treatments. One example is MSNs functionalized with rabies virus glycopeptide (RVG), which were engineered for antiviral drug delivery, particularly targeting neurotropic virus infections. The RVG peptide facilitates specific interactions with neuronal cells, improving drug transport across the BBB [37]. In cancer therapy, MSNs modified with a selenol-modified uPA-specific peptide were employed for targeted resveratrol delivery in triple-negative breast cancer. This peptide enhances protease-mediated drug release, improving tumor selectivity and therapeutic efficacy [67]. Additionally, MSNs were functionalized with titanium-binding peptides (TiBP1, TiBP2) to improve osteointegration and bioactivity in titanium-based implants. These biomaterial-functionalized nanoparticles facilitate bone regeneration and implant stability, enhancing their applications in orthopedic and dental medicine [35].

### 3.5. Superparamagnetic and Iron Oxide Nanoparticles

Superparamagnetic iron oxide nanoparticles (SPIONs) were functionalized with peptides to enhance their applications in targeted imaging, cancer theranostics, and molecular diagnostics. These nanoparticles offer magnetic responsiveness, making them particularly valuable for MRI, fluorescence imaging, and targeted drug delivery. One notable example is SPIONs conjugated with the cell-penetrating peptide (gH625), which have been studied for use in fluorescence and magnetic imaging in cancer theranostics. The gH625 peptide improves cellular uptake, allowing for enhanced imaging contrast and drug delivery efficiency [73]. For molecular cancer imaging, USPIOs functionalized with galectin-1-targeting peptides (P7) were explored for thyroid cancer imaging, leveraging galectin-1’s role in tumor progression and immune suppression [24]. Additionally, iron oxide nanoparticles (Fe_3_O_4_ NPs) functionalized with glypican-3 ligand peptides (GPC3) were developed for ultrasound imaging and cancer therapy, particularly in hepatocellular carcinoma. These nanoparticles facilitate precise tumor localization while enabling theranostic (therapy + diagnosis) applications [26].

### 3.6. Quantum Dots and Carbon-Based Nanocarriers

Quantum dots (QDs) and carbon-based nanomaterials have been extensively studied as imaging agents and nanocarriers, offering high photostability, tunable fluorescence, and biocompatibility for biomedical applications. These nanomaterials have been functionalized with peptides to improve their targeting specificity, drug delivery efficiency, and theranostic capabilities. CdSe/ZnS QDs functionalized with peptides containing D-penicillamine and histidine were employed for the fluorescence monitoring of cancer treatments in melanoma cells. This peptide-QD system enables real-time imaging of therapeutic response, improving treatment assessment and monitoring [25]. Graphene oxide quantum dots (GQDs) modified with the tumor-homing F3 peptide were developed for targeted breast cancer imaging and therapy. The F3 peptide enhances tumor accumulation, allowing for high-contrast imaging and precise drug delivery [49]. In carbon-based nanocarriers, multiwalled carbon nanotubes (MWCNTs) functionalized with the TAT peptide were explored for cancer-targeted drug delivery, leveraging TAT’s ability to facilitate cellular uptake via receptor-independent translocation [52]. Additionally, functionalized carbon nanotubes (f-CNTs) conjugated with the antimicrobial LL-37 peptide were designed as a dual drug delivery system for doxorubicin and LL-37, enabling cancer therapy and infection control to be implemented simultaneously [89].

### 3.7. Hydrogel- and Biomaterial-Based Peptide Functionalization

PF hydrogels and biomaterials have been extensively developed for regenerative medicine, drug delivery, and antimicrobial applications. These peptide-modified materials improve bioactivity, tissue regeneration, and therapeutic efficiency, making them valuable in wound healing, orthopedic implants, and infection prevention. One key example is the RADA16 nanofiber hydrogel functionalized with a copper peptide (GHK), which has been employed for wound healing in diabetic patients. The GHK peptide promotes collagen synthesis, angiogenesis, and tissue repair, facilitating faster wound recovery and improved skin regeneration [90]. In orthopedic and dental implants, polyetheretherketone (PEEK) functionalized with osteogenic growth peptide (OGP) has been developed to enhance osteogenesis. OGP stimulates bone cell differentiation and mineralization, improving osseointegration and bone regeneration in implant applications [91]. Titanium-based biomaterials have also been modified to improve their bioactivity and infection resistance. Titanium implant surfaces functionalized with TiBP1 and TiBP2 demonstrated enhanced osteointegration and bioactivity, supporting bone–implant interactions and long-term implant stability [35]. Additionally, to combat biomaterial-associated infections, supramolecular coatings on titanium implants modified with the antimicrobial peptide SAAP-148 were developed. SAAP-148 exhibits potent antibacterial activity against multidrug-resistant pathogens, preventing implant-related infections and improving post-surgical outcomes [34].

Table 2 explores the diverse peptide-functionalized nanomaterials, emphasizing their structural variations, functionalization strategies, and biomedical applications. Gold-based nanomaterials, widely used in cancer therapy and imaging, are functionalized with tumor-targeting peptides (e.g., RGD, iRGD) and amyloid-targeting peptides for Alzheimer’s treatment. Polymer-based carriers, known for their biodegradability and controlled drug release, incorporate RGD peptides, tumor-homing peptides, and selective cell-penetrating peptides for cancer therapy and regenerative medicine. Lipid-based systems leverage T7, α-NTP, and curcumin-binding peptides for drug delivery across biological barriers. Silica, metal, and quantum dots integrate antimicrobial and imaging peptides for biosensing and theranostic applications. Carbon-based nanomaterials enhance drug delivery and diagnostics through cell-penetrating and antibacterial peptides. This table provides a comprehensive reference for peptide-functionalized nanomaterials in nanomedicine.

## 4. Biomedical Applications of PF Products

### 4.1. Cancer Therapy

PF nanoparticles have been employed in cancer treatment to enhance targeted drug delivery, improve tumor penetration, and reduce systemic toxicity. These strategies enable precise drug release, increased accumulation at tumor sites, and minimized off-target effects, ultimately improving therapeutic outcomes.

**Figure 3:**
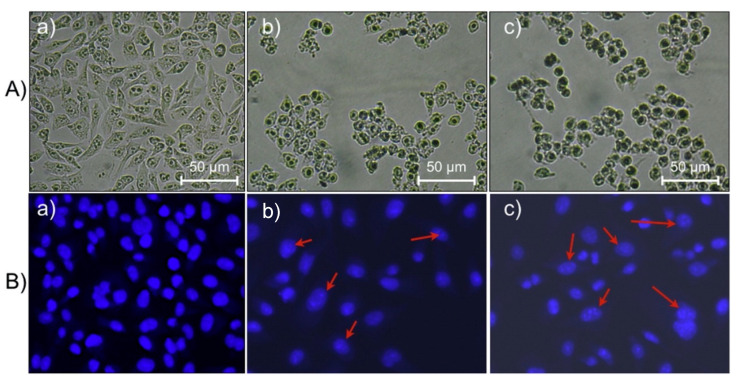
Microscopic investigation and morphological changes in HeLa cells treated with CPCN NPs loaded with DOX: (**A**) optical microscopy images; (**B**) fluorescence microscopy images of cells stained with DAPI; (**a**) control cells, and (**b**,**c**) NP-treated cells at 1.0 and 2.0 μg/mL, respectively. Arrows indicate fragmented DNA in apoptotic cells [5].

Collagen PF chitosan nanoparticles have been developed to optimize DOX delivery to tumors, exhibiting pH-responsive behavior that ensures drug release occurs in the acidic tumor microenvironment. This enhances therapeutic efficacy while reducing systemic toxicity. Studies have demonstrated their high encapsulation efficiency and anti-proliferative effects against HeLa cells while maintaining their biocompatibility with normal cells (Figure 3) [5]. Similarly, LinTT1 PF liposomes, co-loaded with DOX and sorafenib, target hypoxic tumor regions in TNBC. Functionalization with the p32-binding LinTT1 peptide improves drug accumulation in tumor-associated macrophages, promoting deeper penetration into hypoxic tumor cores. Comparative studies indicate higher drug retention in 3D tumor spheroids than non-functionalized liposomes, leading to better treatment responses [7].

In melanoma therapy, intrinsically radiolabeled gold nanoparticles conjugated with cyclic RGD peptides enable tumor-specific localization. These nanoparticles accumulate in melanoma tumors through receptor-mediated interactions, reducing radiation exposure to healthy tissues while enhancing treatment efficacy. Tumor regression has been observed with these nanoparticles without significant body weight loss or systemic toxicity [2].

Beyond improving drug delivery, PF nanoparticles help overcome drug resistance and reduce tumor metastasis. Multivalent PF gold nanorods, designed for siRNA delivery, inhibit epithelial-to-mesenchymal transition (EMT), a critical process in metastasis. By silencing the Notch1 gene, these nanoparticles reduce cancer cell invasiveness and prevent metastasis in breast cancer models, with in vivo studies confirming the reduced tumor spread [15]. For drug-resistant tumors, PEG-PLA nanoparticles functionalized with tumor-homing peptide F3 have been explored for chemo-photodynamic combination therapy, increasing tumor accumulation and cytotoxicity. Functionalization with the F3 peptide enhances tumor penetration, resulting in greater therapeutic efficacy compared to chemotherapy alone [9]. Additionally, gambogic acid-loaded nanostructured lipid carriers (NLCs) modified with cyclic RGD and RGERPPR peptides exhibit increased tumor targeting in breast cancer models. Cellular uptake studies reveal heightened cytotoxic effects on cancer cells, with in vivo imaging confirming enhanced nanoparticle accumulation at tumor sites [11].

### 4.2. Neurodegenerative Disease Treatment

PF nanoparticles have been explored as therapeutic and diagnostic tools for Alzheimer’s disease (AD), focusing on Aβ aggregation inhibition, early diagnosis, and non-invasive interventions. These nanoparticles use specific peptide interactions to bind, detect, or disaggregate toxic Aβ aggregates, offering potential for improved AD management.

Phosphatidic acid- and apolipoprotein E (ApoE)-derived PF liposomes have been developed to bind Aβ aggregates, reducing their formation and promoting disaggregation. Capable of crossing the BBB, these liposomes exhibit a 70% inhibition of Aβ aggregation after 72 h and a 60% reduction in preformed aggregates over 120 h. Studies indicate five-fold greater BBB permeability compared to non-functionalized liposomes, supporting their potential as an Aβ-targeted therapy for AD [18]. Another strategy involves cysteine-amyloid-beta PF gold nanoparticles, which serve both diagnostic and therapeutic purposes. These nanoparticles detect subfemtomolar concentrations of Aβ peptides through spectral changes, facilitating early diagnosis. Additionally, they redirect aggregation pathways, reducing the formation of toxic oligomers and fibrils [19].

Beyond aggregation inhibition, PF gold nanostructures have been investigated for photothermal therapy of Aβ aggregates. These nanoparticles bind to Aβ and, upon near-infrared (NIR) irradiation, generate localized heat, selectively disrupting toxic aggregates. In vitro studies indicate that small gold nanospheres (1.4 nm) significantly affect BBB integrity, with their surface charge influencing BBB passage. Functionalization with poly(ethylene glycol) (PEG) improves stability and permeability, optimizing their use for AD treatment [95]. Additionally, Prussian blue nanoparticles functionalized with CKLVFFAED peptides offer both antioxidant effects and Aβ plaque reduction. These nanoparticles mimic antioxidant enzymes to neutralize reactive oxygen species (ROS) while their photothermal properties facilitate Aβ fibril dissociation under NIR light. In vivo studies show reduced Aβ plaque deposition and decreased neuroinflammation in AD models, highlighting their neuroprotective potential [115].

### 4.3. Inflammatory Disorders

PF nanoparticles have been explored in inflammatory and autoimmune disease treatments to enhance drug retention, reduce toxicity, and improve therapeutic outcomes. These approaches have been investigated for conditions such as rheumatoid arthritis (RA), osteoarthritis (OA), and skin inflammation, offering the targeted and sustained delivery of bioactive molecules.

For osteoarthritis treatment, functionalized nanogels incorporating endothelin-1 and bradykinin receptor antagonists have been designed to modulate inflammatory pathways and prevent cartilage degradation. These nanogels utilize chitosan functionalized with an endothelin receptor antagonist (BQ-123-CHI) and HA functionalized with a bradykinin receptor antagonist (R-954-HA). In an osteoarthritis equine organoid model, this combination therapy reduced inflammatory and cartilage degradation markers while exhibiting biocompatibility with chondrocytes and promoting collagen synthesis, indicating potential for long-term joint protection and pain management [85].

In the treatment of skin inflammation and oxidative stress, cell-penetrating PF liquid crystalline nanodispersions (LCNs) were developed to enhance transdermal drug delivery. These nanoparticles, functionalized with CPPs, improve the skin-permeability of lipoic acid, an antioxidant with anti-inflammatory properties. Following UVB-induced oxidative stress, studies have demonstrated that LCNs significantly lower oxidative stress markers, including reduced myeloperoxidase (MPO) activity and pro-inflammatory cytokine levels (TNF-α and IL-1β), suggesting improved antioxidant activity and enhanced anti-inflammatory effects in topical applications [55].

### 4.4. Infectious Diseases

PF nanoparticles have been explored for the treatment and diagnosis of infectious diseases, providing targeted antimicrobial therapy, controlled drug release, and rapid pathogen detection. These approaches aim to improve efficacy, reduce systemic toxicity, and enhance specificity for bacterial and viral infections.

In orthopedic applications, mesoporous silica nanoparticles loaded with the HHC36 antimicrobial peptide have been developed for sustained bacterial eradication while promoting bone integration. These nanoparticles allow for controlled release of antimicrobial peptides over 30 days, achieving over 95% antimicrobial activity against multiple bacterial strains, including MRSA and *P. aeruginosa*. Additionally, they inhibit bacterial biofilm formation and enhance osteogenic differentiation in bone marrow-derived mesenchymal stem cells, addressing both infection control and bone regeneration challenges in orthopedic implants [29]. Similarly, SAAP-148 PF coatings on titanium implants have been designed to prevent biomaterial-associated infections (BAI) by inhibiting bacterial biofilm formation. These coatings exhibit broad-spectrum activity against antibiotic-resistant strains of *S. aureus*, *E. coli*, and *A. baumannii*, effectively reducing bacterial colonization on implant surfaces. In vivo models confirm significant prevention of bacterial adhesion without inducing toxicity, supporting their potential use in implant protection [34].

**Figure 4:**
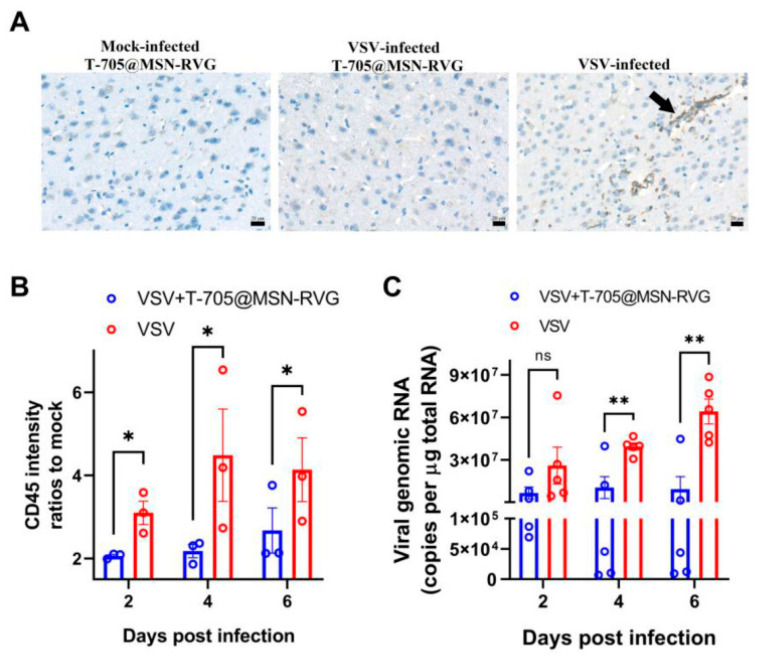
Changes in cell inflammation and viral load post treatment with six-week-old Balb/c mice (VSV-GFP infected at 100 FFU) inoculated with 25 μL of T-705@MSN-RVG (2 mg/mL) via IV route. (**A**) Shows the CD45-positive cells in mouse brains visualized in the mock-infected T-705@MSN-RVG-inoculated group, VSV-infected T-705@MSN-RVG therapy group, and VSV-infected virus control group. Scale bar 20 μm. The black arrow indicates the CD45-positive cells with dark gray staining in the cells. (**B**) Shows the CD45 intensity ratios to mock and (**C**) viral genomic RNA (copies/μg total RNA) of the virus control group and VSV+T-705@MSN-RVG therapy group, analyzed at 2, 4, and 6 dpi. Statistical analysis of grouped comparisons was carried out via Student’s *t*-test (ns represents not significant, * *p* < 0.05; ** *p* < 0.01). The bar graph represents means ± SE [37].

PF nanoparticles have also been investigated for antiviral applications, including targeted drug delivery and broad-spectrum viral inhibition. Selenium nanoparticles functionalized with antiviral peptides have demonstrated high efficacy against SARS-CoV-2 variants (BA.4, BA.5, and XBB) and respiratory syncytial virus (RSV). In vitro studies indicate that functionalized selenium nanoparticles inhibit SARS-CoV-2 replication by up to 100% within 15 min, outperforming conventional antiviral peptides. These nanoparticles interact with SARS-CoV-2 3CL protease, a key enzyme in viral replication, supporting their use in pandemic-preparedness strategies [36]. For neurotropic virus infections, RVG-functionalized favipiravir-loaded mesoporous silica nanoparticles have been developed to enhance BBB penetration. These nanoparticles improve drug delivery to the central nervous system (CNS), increasing antiviral efficacy against rabies, Zika, and poliovirus. In vivo studies show that nanoparticle treatment reduces viral RNA levels, decreases virus proliferation, and improves survival rates by 77% compared to 23% in non-treated controls, indicating their potential as a targeted therapy for CNS viral infections (Figure 4) [37].

Beyond therapeutic applications, PF nanoparticles have been applied in biosensing and rapid pathogen detection. PF microcantilevers functionalized with peptide ligands were developed to detect *Bacillus subtilis* spores, a biowarfare agent. These biosensors exhibit high binding specificity and enable real-time pathogen detection, providing a non-antibody-based platform for rapid biodefense applications [8]. Additionally, biosensor-based viral detection systems using PF microcantilevers have been developed for aquatic viral disease monitoring. These systems utilize antimicrobial PF surfaces to detect and bind viral pathogens, allowing for the real-time tracking of viral outbreaks in aquaculture environments. Studies indicate high binding affinity to viral particles, confirming their application in early viral detection and disease management [116].

### 4.5. Regenerative Medicine and Tissue Engineering

PF biomaterials have been developed to enhance tissue integration, wound healing, and implant performance. These materials facilitate bone regeneration, vascularization, and antimicrobial protection, improving outcomes in orthopedic, soft tissue, and neurological applications.

For orthopedic and dental implants, BMP-2 knuckle PF titanium dioxide nanotubes have been designed to enhance bone integration by promoting osteoblast activity and bone formation. Studies indicate that these nanotubes significantly increase bone–implant contact, fluorescence-based bone deposition, and the gene expression of osteogenic markers. The biomaterial modification accelerates osseointegration, supporting its potential use in long-term implant stability [32]. Similarly, RGD-functionalized biomimetic coatings have been developed to improve endothelial cell adhesion, facilitating vascular graft and stent endothelialization. These coatings provide enhanced endothelial cell retention under shear stress, making them beneficial for vascular applications requiring rapid endothelialization [46].

In wound healing applications, GHK-functionalized RADA16 nanofiber hydrogels have been investigated to promote angiogenesis, tissue remodeling, and diabetic wound closure. These self-assembling peptide-based hydrogels mimic natural extracellular matrix nanofibers, facilitating endothelial cell adhesion, neovascularization, and collagen deposition. In diabetic wound models, these hydrogels significantly accelerate wound healing and reduce inflammation, demonstrating their potential as biocompatible scaffolds for chronic wound management [90]. Additionally, dopamine-immobilized antimicrobial peptides integrated into polycaprolactone/sodium alginate fibers have been developed to prevent surgical site infections (SSIs). These engineered microfibers provide broad-spectrum antimicrobial activity and maintain mechanical integrity for suturing applications. Studies indicate that these fibers prevent bacterial colonization for at least 60 h while accelerating wound closure and re-epithelialization, showing superior healing potential compared to conventional sutures [10].

For neurological applications, TAT-functionalized mesoporous silica nanoparticles have been engineered to enhance methotrexate penetration across the BBB. This functionalization improves drug delivery efficiency to glioblastoma tumors, leading to increased therapeutic efficacy and reduced systemic toxicity. These nanoparticles offer a potential solution for targeted brain cancer therapy, addressing limitations in drug transport across the BBB [54].

Table 3 analyzes the physicochemical properties of nanoparticles and their impact on biomedical applications. Size influences biological interactions, with 10–100 nm particles enhancing tumor penetration and 100–250 nm particles prolonging circulation. Surface charge (zeta potential) affects uptake, where positive charges improve cellular entry, and negative charges enhance circulation stability. Encapsulation efficiency (>70%) optimizes drug delivery, while pH- and redox-responsive behaviors enable targeted tumor drug release. Their high binding affinity ensures selective targeting in cancer and neurodegenerative therapies. BBB permeability increases 3–13×, improving CNS drug delivery. Tumor accumulation (>5% ID/g) optimizes therapeutic effects. Multivalent peptides enhance receptor binding (≥3×). Sustained circulation (>12 h) supports extended drug action, while biocompatibility (>80% cell viability) minimizes toxicity. Antimicrobial and antiviral properties exhibit > 95% eradication efficiency.

Table 4 summarizes key nanomedicine applications, focusing on therapeutic benefits, technical advancements, and quantitative outcomes. Cancer therapy utilizes nanocarriers to enhance drug bioavailability, targeted tumor accumulation, and apoptosis induction. Gene therapy achieves high transfection efficiencies and siRNA stability, leading to significant tumor suppression. Advanced imaging (MRI, PET, fluorescence) improves tumor detection, while theranostic platforms integrate therapy and imaging for enhanced cancer management. Neurological applications show increased BBB penetration and neuroprotection in Alzheimer’s and Parkinson’s. Regenerative medicine promotes osteogenesis, wound healing, collagen deposition, and infection resistance. Antibacterial and antiviral nanotherapies demonstrate high pathogen eradication and sustained drug release. Autoimmune and cardiovascular treatments leverage nanotechnology for inflammation reduction and vascular repair. The data highlights the improved bioavailability, targeted delivery, and extended circulation, reinforcing nanomedicine’s potential for precision medicine and optimized therapeutic efficacy.

## 5. Evaluation and Testing of PF Products

### 5.1. Physicochemical Characterization and Stability Analysis

PF nanoparticles undergo rigorous structural characterization to ensure their consistency, stability, and functionality. Techniques such as Fourier Transform Infrared (FTIR), Transmission Electron Microscopy (TEM), Scanning Electron Microscopy (SEM), Dynamic Light Scattering (DLS), and Atomic Force Microscopy (AFM) assess nanoparticle size, surface morphology, and molecular interactions, verifying successful peptide conjugation and uniform particle distribution [5,26,54,65,89,96,110,117].

Peptide functionalization is evaluated through binding affinity assays, molecular dynamics simulations, and surface plasmon resonance studies to measure peptide interactions with target molecules. X-ray Photoelectron Spectroscopy (XPS) and water contact angle analysis further assess surface hydrophilicity and chemical modifications, ensuring optimal functionality for biomedical applications [21,41,75,89,92,119].

To confirm their long-term stability and efficacy, PF materials undergo aqueous stability tests, zeta potential analysis, and stability assessments in human plasma. Drug encapsulation efficiency and pH-responsive drug release are analyzed to ensure controlled, sustained therapeutic delivery [2,40,51,54,95]. These analyses confirm the stability and effectiveness of PF nanoparticles in targeted applications [50,53,71,83,98,118].

### 5.2. Cellular Uptake, Cytotoxicity and Biocompatibility

For safe clinical applications, PF products must exhibit minimal toxicity while effectively interacting with target cells. Cytotoxicity tests, apoptosis assays, and mitochondrial membrane potential analysis can assess their impact on both healthy and cancerous cells [7,13,15,45,49,50,64,70,77,79,100,117]. Reactive oxygen species (ROS) measurements evaluate the oxidative stress induced by these materials.

To confirm biocompatibility, hemolysis assays, osteogenic differentiation studies, and inflammatory response evaluations ensure that PF nanoparticles do not trigger adverse immune reactions [10,29,33,34,56,85].

To verify efficient cellular uptake, confocal microscopy, fluorescence imaging, and receptor-mediated uptake studies assess nanoparticles’ penetration into tumor cells, ability to cross biological barriers, and subcellular distribution, ensuring optimal targeting efficiency [3,4,27,40,46,53,70,73,98].

### 5.3. Cancer Therapy Efficacy and Tumor Targeting

PF nanoparticles play a crucial role in targeted cancer therapy, requiring a thorough evaluation of their effectiveness in drug delivery and tumor selectivity. Tumor spheroid penetration assays, HER2-targeted uptake studies, and prostate-specific membrane antigen (PSMA) binding assays confirm their specificity in reaching tumor cells [6,9,11,12,14,50,60,71,83,84,118].

For in vivo validation, xenograft tumor models are used to test the survival rate and tumor growth inhibition, and assess the biodistribution, determining their ability to shrink tumors and improve patient outcomes [2,9,14,59,60,83,84,86,107,120].

Additionally, near-infrared (NIR) photothermal studies, fluorescence resonance energy transfer (FRET) analysis, and caspase-3 activation assays evaluate whether PF nanoparticles can induce heat-based apoptosis, enhancing photothermal and chemotherapy treatments [58,65,115,121].

### 5.4. Molecular Imaging, Biosensors and Diagnostic Applications

To improve early cancer diagnosis, PF imaging agents are evaluated through MRI contrast imaging, fluorescence lifetime imaging microscopy (FLIM), and PET imaging, enabling real-time tumor monitoring and non-invasive detection [24,27,28,113,119].

PF biosensors contribute to rapid disease detection via microcantilever biosensing, CMOS sensor fabrication, and capacitance-to-digital conversion techniques, aiding in the identification of cancer biomarkers and circulating tumor cells (CTCs) [8,114,116,122].

### 5.5. Antimicrobial and Infection Control Testing

PF materials exhibit strong antimicrobial properties, necessitating evaluation through bacterial growth inhibition assays, DNA cleavage tests, and biofilm disruption studies to determine their effectiveness in pathogen-elimination [10,29,34,38,39].

For biomedical implant applications, bone–implant integration, micro-CT bone formation, and osteoblast proliferation studies assess both their safety and long-term durability [32,78,91].

### 5.6. Neurological and Blood–Brain Barrier Studies

For brain-targeted drug delivery, PF nanoparticles must effectively cross the BBB. BBB permeability assays, glioblastoma targeting studies, and neuroprotection assessments determine their ability to reach the brain and deliver therapeutic payloads [20,21,58,95,104].

For Alzheimer’s disease treatment, Aβ binding assays, neuroinflammation reduction studies, and in vivo plaque reduction experiments evaluate their potential in combating neurodegeneration and supporting neuroprotection [19,68,115].

### 5.7. Inflammatory and Autoimmune Disease Models

PF materials are investigated for their role in autoimmune diseases such as arthritis and diabetes.

For diabetic wound healing and tissue repair, endothelial adhesion assays, collagen deposition studies, and skin penetration tests evaluate their effectiveness in accelerating wound healing and promoting tissue regeneration [56,90].

Table 5 summarizes the key testing and evaluation methods for nanoparticle-based therapies. Physicochemical characterization (DLS, TEM, SEM) ensures their size (50–200 nm), charge (−30 to +40 mV), and stability (>6 months). Functionalization analysis (SPR, molecular docking) confirms >80% binding efficiency under physiological conditions. Cellular uptake studies show 3–10× increased internalization via receptor-mediated endocytosis. In vitro drug release achieves >90% release at pH 5.5 or a high GSH, enhancing bioavailability (3–10×). Cytotoxicity assays confirm >90% cancer cell apoptosis with >80% normal cell viability. In vivo studies validate tumor targeting (>5% ID/g), prolonged retention (24 h), and tumor suppression (>70%). Gene silencing achieves >80% knockdown efficiency. BBB studies show 5–13× enhanced uptake. Regenerative medicine accelerates wound healing (2×), while antimicrobial/antiviral tests confirm >98% bacterial eradication, 100% viral inhibition (SARS-CoV-2). 

## 6. Achievements and Advances in PF Technologies

PF nanoparticles have emerged as highly versatile tools in precision medicine, driving transformative advancements in drug delivery, gene therapy, diagnostics, imaging, regenerative medicine, and antimicrobial/antiviral strategies. Their remarkable physicochemical and biological properties, including pH-responsive drug release, high binding affinity, enhanced cellular uptake, selective tumor accumulation, and bioactivity modulation, have positioned them at the forefront of targeted and personalized therapeutic strategies.

### 6.1. Precision Drug Delivery and Enhanced Therapeutic Efficacy

*PF Nanoparticles for Targeted Drug Release*: A major advantage of PF nanoparticles is their ability to control drug release through precisely tuned physicochemical properties, allowing for enhanced targeting, reduced systemic toxicity, and improved therapeutic outcomes. The ability to release therapeutic agents in response to specific environmental cues, such as pH variations in tumor microenvironments or intracellular compartments, has made them invaluable for cancer therapy and inflammatory disease treatment. For instance, chitosan-based PF nanoparticles (~100 nm) utilize electrostatic interactions to enable pH-sensitive drug release, ensuring high encapsulation efficiency for targeted cancer therapy [5]. Similarly, gold nanocages functionalized with A54 peptides allow for near-infrared (NIR) light-triggered drug release, facilitating precise DOX delivery to tumor sites while minimizing off-target toxicity [83]. In another approach, zeolitic imidazolate framework-8 (ZIF-8) nanocarriers functionalized with TAT peptides exhibit 95% drug release at pH 5.5, effectively inducing 93.5% apoptosis in cancer cells, demonstrating their high therapeutic efficacy [53]. Meanwhile, curcumin-loaded aerogel PF nanoparticles provide stable drug retention at neutral pH but undergo controlled release in acidic conditions, ensuring localized tumor-targeted therapy with minimal systemic side effects [71].

**Figure 5:**
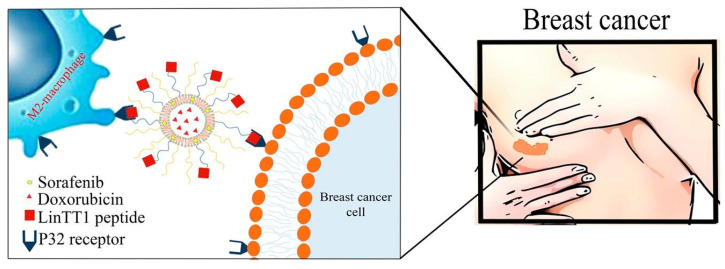
Schematic illustration of the conjugation of LinTT1-functionalized liposomes with DOX and sorafenib (SRF) co-loaded in therapeutic liposomes, designed to target the p32 protein in triple-negative breast cancer cells in both 2D and 3D breast cancer cellular models [7].

*Optimizing Tumor-Specific Drug Accumulation*: Targeted drug delivery approaches have evolved significantly to improve tumor retention, ensuring that therapeutic agents remain localized at the site of action for prolonged effectiveness and minimal systemic toxicity. Liposomes functionalized with LinTT1 peptide have been shown to enhance drug accumulation in hypoxic tumor regions, significantly improving therapeutic outcomes in TNBC (Figure 5) [7]. Additionally, CD44-targeting oil-core polymer nanocapsules [3] and PEG-PLA nanoparticles functionalized with tumor-homing F3 peptide [9] exhibit high tumor accumulation, leading to greater drug retention and improved chemotherapy efficiency. Furthermore, multivalent PF liposomal DOX demonstrated superior chemotherapy selectivity by precisely targeting αvβ6-expressing tumors, thereby reducing systemic toxicity and increasing cancer cell elimination [41].

*Advancing Gene Therapy for Cancer Treatment*: Beyond traditional chemotherapeutics, PF nanoparticles are playing an essential role in gene therapy, where they serve as platforms for enhancing gene silencing, genome editing, and tumor modulation. For example, PF siRNA nanocarriers significantly improve gene silencing efficiency, offering a promising approach to suppressing metastatic cancer progression [15]. Oncolytic adenoviral vectors conjugated with peptide nucleic acids selectively target Bcl-2 gene G-quadruplexes, improving the safety and efficacy of cancer gene therapy [64]. Furthermore, in lung cancer models, hyperbranched poly(amido amine) nanoparticles functionalized with iRGD peptide have shown remarkable tumor-suppression capabilities, delivering redox-responsive siRNA directly to cancerous tissues [16].

*Overcoming Drug Resistance and Enhancing Chemotherapy*: One of the most pressing challenges in oncology is overcoming MDR, which limits the efficacy of conventional chemotherapy. PF-based nanosystems have been engineered to modulate key resistance pathways and enhance drug delivery efficiency, ensuring higher treatment success rates. For instance, stearic acid-modified GE11 PF nanoplatforms (GENPs) have been shown to modulate the EGFR-PI3K/AKT pathway, successfully suppressing TNBC and bone metastases [99]. In addition, HA -TOS nanoparticles functionalized with tumor-penetrating tLyP-1 peptide improve docetaxel delivery, significantly reducing systemic toxicity while enhancing chemotherapy outcomes [69]. Moreover, dual-responsive nanoparticles functionalized with L-peptide enable pH- and temperature-controlled paclitaxel release, demonstrating effective tumor growth inhibition [118].

### 6.2. Overcoming Biological Barriers in Neurological and Neurodegenerative Diseases

*Crossing the BBB for Neurological Drug Delivery*: The BBB poses a major challenge in the treatment of neurological disorders, limiting the transport of therapeutic molecules into the brain. PF nanoparticles have been engineered to bypass this barrier, offering new opportunities for treating Alzheimer’s disease and stroke. Liposomes functionalized with phosphatidic acid and ApoE-derived peptides have successfully penetrated the BBB, leading to reduced amyloid-beta aggregation and enhanced Alzheimer’s disease therapy [18]. Similarly, cysteine-amyloid-beta PF gold nanoparticles (Cys-Abeta@AuNPs) provide ultra-sensitive detection of amyloid-beta, allowing for early-stage Alzheimer’s diagnoses [19].

*Neuroprotective Strategies for Stroke and Alzheimer’s Disease*: PF-based nanocarriers are also being explored for stroke treatment and neuroprotection. Tanshinone IIA-loaded dendrimers functionalized with T7 and PGP peptides exhibit improved BBB penetration, leading to reduced neuroinflammation in ischemic stroke models [20]. Meanwhile, in Alzheimer’s disease models, chitosan nanoparticles functionalized with TG peptide significantly reduce Tau phosphorylation, offering potential neuroprotective benefits and cognitive improvement [22].

### 6.3. Advanced Imaging and Diagnostics for Early Disease Detection

*Precision Imaging for Cancer Detection*: PF-based imaging agents have significantly advanced cancer diagnostics, improving their sensitivity and specificity in molecular imaging applications. For instance, Surface-Enhanced Resonance Raman Scattering (SERRS)-active nanostructures functionalized with PreS1 peptide provide highly sensitive liver cancer detection, while gold nanoparticle aggregates conjugated with cyclic RGD peptides improve colorectal cancer imaging [1,23]. Additionally, USPIO nanoparticles functionalized with galectin-1 peptides have demonstrated 100% specificity in thyroid cancer imaging, helping to reduce unnecessary surgical procedures [24].

*Real-Time Biosensing and Pathogen Detection*: PF-based biosensors enable rapid and highly specific pathogen detection, broadening their applications in infectious disease diagnostics. For example, microcantilever biosensors functionalized with AMP have been utilized to detect grouper nervous necrosis virus (NNV) with high antiviral sensitivity [116]. Similarly, bacterial-binding peptide-based biosensors allow for label-free detection of *B. subtilis*, opening avenues for real-time infection diagnostics [8].

## 7. Limitations, Challenges, and Future Directions of PF Technologies

Despite their transformative potential in drug delivery, gene therapy, imaging, diagnostics, regenerative medicine, and antimicrobial applications, PF nanoparticles face significant challenges that hinder their clinical translation and widespread adoption. These challenges range from biocompatibility and toxicity concerns to scalability, regulatory barriers, and issues related to stability and targeted delivery. Addressing these hurdles is essential for advancing PF technologies toward real-world applications.

### 7.1. Biocompatibility, Safety, and Immune Response Challenges

*Toxicity and Immune System Activation*: While PF nanoparticles offer significant biomedical potential, concerns persist regarding their toxicity and immune activation. Gold nanorods functionalized with RGD/GLF peptides have shown hepatic toxicity and immune response activation, raising safety concerns in liver disease models [76]. Similarly, cationic cell-penetrating PF gold nanoparticles exhibit high cellular penetration but may cause off-target cytotoxicity due to non-specific uptake by healthy tissues [70]. Immune responses to PF nanocarriers remain a challenge, as seen with human serum albumin (HSA)-based glypican-3-targeting nanoparticles and liposomal DOX formulations, which may reduce circulation time and therapeutic efficacy [40,41]. Oncolytic adenoviral vectors conjugated with PF molecules also require further optimization to mitigate immune-triggered cytotoxicity, limiting clinical utility [64].

*Long-Term Safety and Biodegradability*: The long-term biocompatibility and clearance of PF nanoparticles remain underexplored. Multiwalled carbon nanotubes functionalized with TAT peptide enhance intracellular drug delivery but may accumulate over time, raising concerns about chronic toxicity [52]. Superparamagnetic iron oxide nanoparticles (SPIONs) used for thyroid cancer imaging require further assessment to evaluate the metal accumulation risks [24]. Polyethyleneimine-based siRNA carriers, while effective in gene therapy, need modifications to improve their biodegradability and reduce their systemic toxicity [42].

*Enhancing Biocompatibility and Safety*: Strategies such as PEGylation, lipid coatings, and hybrid organic–inorganic nanocarriers aim to extend the circulation time and reduce immune recognition. PEG-PLA nanoparticles help lower immune responses and enhance stability [9], while HA-TOS nanoparticles functionalized with PEG offer improved safety by minimizing immune reactions [69]. Stimuli-responsive, biodegradable nanocarriers like pH-sensitive resveratrol-loaded silica nanoparticles are being designed to degrade in disease-specific environments, reducing off-target effects and systemic accumulation [67].

### 7.2. Stability, Peptide Integrity, and Degradation Challenges

Peptides in PF-based systems face enzymatic degradation in biological environments, leading to a short half-life and reduced therapeutic effectiveness. This necessitates modifications to enhance their bioavailability. PF liposomes and zinc oxide nanoparticles, for instance, experience significant degradation in biological fluids, limiting their long-term applicability [18,111]. Similarly, LinTT1-functionalized liposomes and oil-core polymer nanocapsules degrade rapidly, reducing their therapeutic bioavailability [3,7].

To address these stability challenges, researchers are exploring polymer encapsulation, PEGylation, and lipid stabilization. Chitosan-functionalized nanogels require improved formulations to enhance their stability and retention at target sites [85]. Likewise, pH-sensitive aerogel particles functionalized with PLP peptides need optimization to regulate their curcumin release kinetics, ensuring sustained and effective drug delivery [71].

### 7.3. Scalability, Manufacturing Hurdles, and Reproducibility

The complex synthesis of PF nanoparticles increases production costs and limits large-scale manufacturing. PEG-PLA nanoparticle formulations, PF titanium implants, and PLGA-T7 nanoparticles require precisely controlled synthesis conditions, making industrial-scale production challenging [9,29,45]. Electrospun PF meshes for lung models and self-assembled PF porous silicon nanovectors also need improved batch-to-batch reproducibility to meet clinical reliability standards [47,51].

To address these challenges, researchers are developing cost-effective, scalable synthesis methods that ensure high-yield production while maintaining reproducibility. Microfluidic-based techniques could enhance batch consistency for biosensors and imaging nanoparticles, such as USPIO conjugates for thyroid cancer imaging [24]. Similarly, standardized synthesis protocols for PET imaging peptides are being explored to improve clinical reliability [119].

### 7.4. Regulatory Barriers, Clinical Translation, and Standardization

Despite the promising preclinical results, many PF nanomedicine platforms remain stalled in early-phase trials due to the limited pharmacokinetic and toxicity data. PROCR-targeting MoS_2_ nanosensors for TNBC detection, Pep-1 liposomes for glioma therapy, and iRGD-siRNA therapy require extensive validation before regulatory approval [4,16,86]. Similarly, CRISPR-Cas9 delivery platforms demand rigorous safety assessments to ensure precise gene-editing outcomes without off-target mutations [17].

To bridge the gap between preclinical and clinical applications, PF nanoparticles must undergo thorough long-term pharmacokinetic and immunogenicity studies. LinTT1-liposomes and iRGD-siRNA therapy require comprehensive toxicity evaluations before advancing to human trials [7,16]. Standardized protocols for glioma-targeting Pep-1 liposomes and gene therapies using TAT-AT7 PF nanoparticles are also essential for FDA compliance and regulatory approval [21,86].

### 7.5. Future Directions: Innovations and Emerging Strategies

Next-generation PF-based therapeutics aim to integrate stimuli-responsive drug release and multifunctional theranostic capabilities. pH-, enzyme-, and light-responsive nanocarriers enable site-specific drug release, enhancing therapeutic precision [71]. Gold nanocages functionalized with photothermal agents offer simultaneous cancer therapy and hyperthermia treatment, improving their overall efficacy [83].

Artificial intelligence (AI) is transforming drug screening and personalized medicine by enabling the real-time optimization of therapeutic strategies. Upconversion@polydopamine (UCNP@PDA) nanoparticles, which consist of an upconversion nanoparticle (UCNP) core for luminescence-based sensing and a polydopamine (PDA) shell for fluorescence resonance energy transfer (FRET) and drug loading, show promise for real-time drug response monitoring. When combined with AI-driven imaging, these multifunctional nanoparticles could enhance personalized treatment approaches [121]. Wearable PF biosensors, such as MXene-based smart bandages, may also allow for continuous real-time infection monitoring, improving early disease detection [114].

PF nanoparticles hold potential for antiviral and antimicrobial applications, necessitating large-scale validation. Selenium-based antiviral nanoparticles require further testing to assess their effectiveness against emerging viral threats [36]. Additionally, antimicrobial coatings for medical implants, such as SAAP-148 titanium implants, should be prioritized for commercial deployment to reduce post-surgical infections [34].

While various peptide-functionalized nanocarrier systems have demonstrated remarkable potential in drug delivery and diagnostics, nucleopeptides present an emerging avenue worth exploring. Nucleopeptides, which integrate nucleobases with peptide backbones, exhibit unique self-assembly properties and strong interactions with biomolecules, making them promising candidates for enhancing nanocarrier functionality. Their ability to form hydrogels and nanostructures could facilitate controlled drug release, while their affinity for nucleic acids and proteins enables targeted gene delivery and diagnostic applications [124]. Additionally, research by Prof. Domenica Musumeci and others has highlighted the potential of nucleopeptides in binding to biomolecules and modulating protein interactions, opening up new possibilities for multifunctional nanocarriers [125]). Future investigations should focus on integrating nucleopeptides into existing nanocarrier platforms to assess their stability, bioavailability, and therapeutic efficacy in drug delivery and molecular diagnostics.

Table 6 comprehensively explores key advancements and challenges in nanomedicine, focusing on stability, drug delivery, targeting strategies, and clinical translation. It highlights nanoparticle stability, encapsulation efficiency, and BBB penetration, showing promising results in controlled drug release and disease-specific targeting. Multimodal therapies and responsive nanomedicine demonstrate potential for enhanced cancer treatment and biosensing, while antimicrobial and autoimmune applications emphasize precision medicine. Emerging fields like neurodegeneration and theranostics could use AI integration and biodegradable platforms for improved efficacy. Future research should optimize scalability, real-time monitoring, and adaptive delivery for personalized and next-generation treatments.

## 8. Conclusions

Peptide-functionalized nanocarriers represent a paradigm shift in precision therapeutics and diagnostics, integrating targeting specificity, bioactive functionality, and modular adaptability into nanomedicine. Their ability to enhance drug delivery efficiency, imaging contrast, and disease-targeted interventions underscores their potential for advancing treatment strategies in oncology, neurology, immunology, and infectious disease management. However, the translation of these platforms into clinical practice remains hindered by peptide stability, immunogenicity, large-scale manufacturing constraints, and regulatory uncertainties. Overcoming these challenges necessitates rational peptide design, the incorporation of enzyme-resistant modifications, scalable production techniques, and harmonized regulatory frameworks to ensure their safety and efficacy.

Future research should focus on multimodal peptide–nanocarrier platforms, integrating stimuli-responsive functionalities, combination therapies, and AI-driven peptide screening to optimize therapeutic performance. The synergy between materials science, bioengineering, and computational peptide design will be critical in accelerating the clinical translation of these technologies. Furthermore, expanding applications beyond oncology—into neurodegenerative disorders, autoimmune diseases, and regenerative medicine—will unlock new frontiers in precision medicine. As peptide-functionalized technologies continue to evolve, their convergence with next-generation biomaterials, personalized medicine, and real-time biosensing will redefine the landscape of targeted therapy, diagnostics, and disease monitoring, paving the way for a new era of nanomedicine.

## Figures and Tables

**Table 1:** Bioactive peptides in targeted drug delivery and therapeutic applications.

Peptide Category	Peptide Examples	Applications/Findings	Ref.
Antimicrobial Peptides (AMPs)	Antimicrobial peptides (AMPs), antimicrobial peptide (HHC36), antimicrobial peptide (SAAP-148), antimicrobial peptide (GLFVDK-Cy7)	AMPs immobilized on biomaterials enhance wound healing, prevent biofilm formation, and combat antibiotic-resistant bacteria. Functionalized titanium implants and nanotherapy approaches show sustained antimicrobial activity and promote tissue regeneration.	[10,29,34,39]
Cell-Penetrating Peptides (CPPs)	Tumor-homing and penetrating peptide (F3), cell-penetrating peptides (cRGD, RGERPPR), TAT-NLS peptide, TAT-AT7 peptide, TAT peptide, tumor-penetrating peptide (tLyP-1), penetratin (Pen)	CPPs enhance targeted drug delivery, improving therapy for drug-resistant cancers, glioma, and inflammatory conditions. Functionalized nanocarriers, nanoparticles, and frameworks aid in genome-editing, blood–brain barrier penetration, and photothermal therapy.	[9,11,17,21,52,53,54,55,56,58,59,69,70,71,72,73]
Functional Peptides for Drug Carrier Enhancement	Fusion peptide (RGD + R(8)), peptide-functionalized siRNA, T7-functionalized peptide, DNP functionalized peptide, thiol-functionalized RGD peptide	Functional peptides improve drug delivery by enhancing cancer treatment, gene silencing, and immunotherapy. Hybrid nanoparticles, gold nanorods, and peptide-functionalized carriers aid in reversing drug resistance, targeted co-delivery, and cell adhesion studies.	[13,15,48,74,75]
Integrin-Targeting Peptides	Cyclic RGD peptide, cell-penetrating peptides (cRGD, RGERPPR), fusion peptide (RGD + R(8)), internalizing-RGD (iRGD), RGD-TAMRA peptide, RGD and GLF tripeptides	Integrin-targeting peptides enhance cancer therapy, drug delivery, and tissue engineering. Functionalized gold nanoparticles, polymeric carriers, and biomimetic coatings improve tumor targeting, vascular grafts, and implant integration.	[1,2,11,13,16,31,46,47,48,76,77,78,79,80]
Organ-Specific Targeting Peptides	Joint-homing peptide (ART-1), liver cancer-targeting peptide, organelle-targeted peptides, adipose homing peptide (AHP)	Organ-specific targeting peptides improve precision drug delivery and imaging. Applications include arthritis therapy, liver cancer treatment, organelle-directed chemotherapy, and obesity-targeted imaging.	[33,65,66,81]

**Table 2:** Key peptide-functionalized nanomaterials and their biomedical applications.

Nanomaterial Category	Examples of Different Structures	Peptides Used in Functionalization	Ref.
Gold-Based	Gold Nanoparticles, Gold Nanorods, Gold Nanostars, Gold Nanoshells, Gold Nanocages	Cyclic RGD peptide, peptide-functionalized siRNA, cysteine–Abeta peptide, PreS1 peptide, antimicrobial peptide (GLFVDK-Cy7), A54 peptide, RGD and GLF tripeptides, cell-penetrating peptide (CPP), PreS1 (21–47) peptide, CRGDK and PMI (p12) peptides, liver cancer-targeting peptide, prohibitin (PHB)-targeting peptide, glypican-3-binding peptide, GFD and SMB peptides, penetratin (Pen) peptide, β-amyloid-specific peptide, peptide dendrimer, colorectal cancer-targeting peptides	[1,2,15,19,23,39,58,62,65,70,76,82,83,92,93,94,95,96,97]
Polymer-Based	Chitosan Nanoparticles, Polysaccharide-Based Nanoparticles, PEG-PLA Nanoparticles, PLA-Based Nanoparticles, Polymersomes	Collagen peptide, IVS4 peptide, tumor-homing and -penetrating peptide (F3), CF peptide, internalizing-RGD (iRGD), T7 and PGP peptides, TAT-AT7 peptide, TG peptide, endothelin-1 and bradykinin receptor antagonist peptides (BQ-123, R-954), tumor-penetrating peptide (tLyP-1), K237 peptide, transferrin-binding peptide (TBP), fibronectin-derived RGD peptide, iNGRt peptide (CRNGR), GE11 peptide, CRGDK peptide, A6 peptide, selective cell-penetrating peptide (SCPP), cyclic RGDfK peptide, WSC02 peptide, CD138-targeting peptide, alpha(3) integrin-binding eptide	[5,6,9,12,16,20,21,22,46,59,60,69,77,79,80,84,85,98,99,100,101,102,103]
Lipid-Based	Liposomes, Nanostructured Lipid Carrier, Lipid Nanoparticles	LinTT1 peptide, H2009.1 tetrameric peptide, cell-penetrating peptides (cRGD, RGERPPR), modified ApoE-derived peptide, joint-homing peptide (ART-1), Pep-1 peptide, Pep-1 and folic acid (FA) peptides, NFL-TBS.40-63 peptide, α-NTP peptide, ATF(24) peptide, P563 peptide, curcumin–lipid ligand, QLPVM peptide	[7,11,18,33,41,68,86,87,88,104,105,106,107,108]
Silica-Based	Mesoporous Silica Nanoparticles, Porous Silicon Nanovectors, Graphene Quantum Dots in Silica	Antimicrobial peptide (HHC36), rabies virus glycopeptide (RVG), selenol-modified uPA-specific peptide, TAT peptide, F3 peptide, tumor-homing peptide (CooP), peptide–silicon nanowires, legumain-responsive peptide	[29,37,49,51,54,67,109,110]
Metal-Based	Superparamagnetic Iron Oxide Nanoparticles, Selenium Nanoparticles, Zinc Oxide Nanoparticles	Galectin-1-targeting peptide (P7), glypican-3 ligand peptide (GPC3), NapFFTLUFLTUTEKKKK, NapFFMLUFLMUMEKKKK, NapFFSAVLQSGFKKKK, TAT peptide, PLAC-1-targeting peptide (GILGFVFTL), TumorFisher Peptide, HER2-targeting peptide, cell-penetrating peptide (gH625)	[24,26,36,53,61,73,111,112]
Quantum Dots	CdSe/ZnS Quantum Dots, Graphene Quantum Dots, NaGdF(4) Nanodots	Peptide with D-penicillamine and histidine, LTVSPWY peptide, PLAC-1 targeting peptide (GILGFVFTL), F3 peptide, CXCR4-antagonistic peptide, adipose homing peptide (AHP)	[25,27,44,49,66,113]
Carbon-Based	Multiwalled Carbon Nanotubes, Functionalized Carbon Nanotubes, MXene-Based Biosensors	TAT peptide, LL-37 peptide, sortase A-targeting peptide	[52,89,114]

**Table 3:** Physicochemical properties of PF nanocarriers in biomedical applications.

Physicochemical Property	Key Features and Quantitative Data	Ref.
Nanoparticle Size (10–250 nm range)	Smaller particles (~10–100 nm) show enhanced tumor penetration, while larger particles (~100–250 nm) improve circulation time. Examples: Gold NPs (~12.5 nm), Liposomes (~100 nm), Iron Oxide NPs (~10 nm), Mesoporous Silica NPs (~79 nm), Micelles (~110 nm).	[2,3,5,9,10,29,59,69,77,82,83,84,98,117]
Zeta Potential (Surface Charge, −30 to +40 mV)	Positively charged particles (+20 to +40 mV) improve cellular uptake; negatively charged particles (−10 to −30 mV) enhance circulation stability. Examples: PEG-PLA NPs (+27.4 mV), Gold NPs (−12.5 mV), Chitosan NPs (+18.7 mV), Liposomes (−21.8 mV).	[9,12,21,33,45,84,98,100]
High Drug Encapsulation Efficiency (>70%)	Common in liposomal, polymeric, and silica-based nanoparticles, ensuring sustained drug release. Examples: Liposomes (92%), PLGA NPs (87%), Micelles (82.5%), Mesoporous Silica NPs (93%), PF Gold NPs (89.8%).	[5,7,11,12,21,69,100,106,118]
pH-Responsive Behavior (Tumor pH ~6.5, Endosomal pH ~5.5)	pH-sensitive drug release enables targeted therapy, especially in tumor microenvironments. Examples: Gold Nanocages (95% drug release at pH 5.5), PLGA NPs (pH 6.5), Liposomes (90% release at pH 5.5).	[12,21,53,71,77,100,118]
Redox-Responsive Drug Release (GSH-Triggered)	Triggered release in cancer cells due to high glutathione (GSH) levels (~10 mM in tumors vs. ~1 mM in normal cells). Examples: Mesoporous Silica NPs (93.5% drug release), Gold NPs (90%), Polymer Micelles (89%).	[21,53,77,118]
High Binding Affinity (Kd < 1 µM, Strong Peptide–Target Interactions)	Ensures precise targeting of cancer cells and amyloid-beta aggregation inhibition. Examples: Amyloid-beta peptide binding (Kd = 0.6 µM), Integrin αvβ3 Binding (Kd = 250 nM), HER2 Binding (Kd = 220 nM).	[1,2,18,19,40,62,92,96]
Increased BBB Permeability (3–13× Higher Penetration)	PF nanoparticles improve drug delivery to the brain. Examples: Liposomes (5× higher BBB permeability), Gold NPs (13-fold higher brain accumulation), Graphene Quantum Dots (4.8× increased BBB crossing).	[4,18,19,52,57,58,95,104,105]
High Tumor Accumulation (>5% ID/g in Tumor Tissue)	Targeted nanoparticles accumulate in tumors, reducing off-target effects. Examples: Gold NPs (8.7% ID/g at 4 h), Iron Oxide NPs (75% tumor sensitivity), Liposomes (50% uptake by M2 macrophages).	[2,7,9,14,26,40,62,69]
High Fluorescence or Imaging Contrast (Strong NIR, PET, MRI Signals)	Used for bioimaging, including fluorescence, MRI, PET, and ultrasound. Examples: Iron Oxide NPs (100% specificity in tumor detection), Gold NPs (over 80% sensitivity), Quantum Dots (35% photoluminescence quantum yield).	[23,24,25,26,27,28,40,92,94,112]
Multivalent Peptide Presentation (≥2× Increased Binding Affinity)	Enhances receptor targeting and cell binding for cancer therapy. Examples: RGD-functionalized NPs (2.5× stronger binding), Tetrameric Peptides (6× increased cytotoxicity), Multivalent Gold NPs (3× increased cellular uptake).	[1,41,93,94]
Selective Cellular Uptake (≥3× Higher in Target Cells vs. Non-Target Cells)	PF nanoparticles show significantly increased uptake in target cells compared to controls. Examples: ZnO NPs (4.8× uptake in PLAC-1 cells), Micelles (3.3× increased tumor accumulation), Liposomes (3.7× higher uptake).	[3,9,21,70,84,86,111,117]
Long Circulation Time (>12 h in vivo, Delayed Clearance)	Improves nanoparticle stability and tumor targeting. Examples: PEGylated Liposomes (24 h half-life), PLGA NPs (18 h circulation time), Peptide-NPs (16 h blood retention).	[2,33,45,60]
Enhanced Apoptosis Induction (>50%)	PF nanoparticles significantly increase cancer cell apoptosis rates. Examples: ZnO NPs (93.5% apoptosis), PLGA NPs (50%+ apoptosis in TNBC), Gold NPs (70% apoptosis induction).	[12,45,67,79,106,111,118]
Increased ROS Production and Mitochondrial Dysfunction (Tumor-Specific Oxidative Stress Induction)	Enhances cancer cell death via oxidative stress. Examples: Micelles (89% ROS production), Gold NPs (92% mitochondrial dysfunction), Iron Oxide NPs (80% cell necrosis).	[11,16,77,79]
Superior Biocompatibility (>80% Cell Viability in Normal Cells, Low Cytotoxicity to Non-Target Cells)	PF nanoparticles minimize toxicity in normal cells while targeting cancer cells. Examples: Gold NPs (>90% biocompatibility), Liposomes (85% cell viability), Graphene QDs (>87%).	[6,10,59,70,79,82,84,100]
Targeted Antibacterial and Antiviral Activity (>95% Eradication Rate)	PF nanoparticles effectively eliminate bacteria and viruses. Examples: Selenium NPs (100% inhibition of Omicron XBB and RSV), Gold NPs (>95% bacterial eradication), Mesoporous Silica NPs (98.8% in vivo bacterial clearance).	[10,29,36,37,38,39,116]

**Table 4:** Therapeutic and diagnostic applications of PF nanocarriers.

Application Area	Specific Purpose (Drug Active or Therapeutic Use)	Key Features and Quantitative Data	Ref.
Cancer Therapy	Targeted chemotherapy (DOX, Paclitaxel, Gemcitabine, Curcumin, etc.)	Tumor accumulation (>5% ID/g), high apoptosis induction (>50%), increased drug bioavailability (up to 10×), pH-responsive drug release (~90% at pH 5.5), and extended circulation time (>12 h).	[1,5,6,7,9,21,41,45,59,69,70,77,84,86,99,100,118]
Gene Therapy	siRNA, CRISPR, and genetic modulation for cancer and other diseases	High gene transfection efficiency (>80%), enhanced siRNA stability (3× longer half-life), gene knockdown (>70%), tumor suppression (>60%), and minimal toxicity to healthy cells.	[12,15,16,17,21,42,74]
Cancer Imaging and Theranostics	Fluorescence, MRI, PET, and photothermal imaging for tumor detection and treatment	Gold NPs (8.7% ID/g at 4 h), iron oxide NPs (75% tumor detection sensitivity), quantum dots (35% photoluminescence quantum yield), and high contrast (NIR/PET/MRI).	[2,19,23,24,25,26,27,28,44,61,62,92,94,112]
Neurological Disorders (Alzheimer’s, Parkinson’s, Stroke)	Targeted therapy for amyloid-beta aggregation inhibition and neuroprotection	Amyloid-beta aggregation inhibition (70%), blood–brain barrier penetration (5× higher), enhanced neuronal survival (>80%), amd significant reduction in neuroinflammation.	[4,18,19,37,42,57,95,104,105]
Osteoarthritis and Orthopedic Therapy	Regenerative therapy and osseointegration for bone and joint diseases	High biocompatibility (>85% cell viability), enhanced bone-implant contact (~50% increase), and increased osteogenesis markers (ALP, Collagen-I upregulated).	[10,29,32,78,85,91]
Wound Healing and Tissue Regeneration	PF biomaterials for enhanced healing and antimicrobial protection	Collagen deposition (>50% increase), angiogenesis stimulation, infection reduction (>95%), enhanced wound closure (2× faster).	[10,30,90]
Antibacterial and Antiviral Therapy	Targeted inhibition of bacterial infections and viruses (SARS-CoV-2, RSV, etc.)	Bacterial eradication (>98%), viral inhibition (100% inhibition of Omicron XBB and RSV), sustained antimicrobial release (>30 days), and minimal host cytotoxicity.	[10,29,36,37,38,39,116]
BBB Penetration	Improved delivery of drugs and genes for brain disorders	BBB permeability enhancement (3–13× higher), improved brain-targeted drug retention (up to 24 h), and neuronal protection (>80%).	[4,52,54,57,58,104]
Inflammatory and Autoimmune Diseases	Targeted therapy for rheumatoid arthritis and inflammatory skin diseases	Joint-specific drug delivery via ART-1 peptide-functionalized liposomes for arthritis treatment (subcutaneous route); enhanced skin penetration using CPP-modified nanodispersions for inflammatory skin therapy. Key outcomes include targeted drug accumulation at disease sites, reduction of inflammatory cytokines (e.g., TNF-α, IL-1β), and significant disease suppression in preclinical models.	[33,55]
Cardiovascular Therapy	Vascular targeting, endothelial regeneration, and imaging	Enhanced endothelial cell proliferation (>60%), increased vascular graft retention (>80%), and improved vascular repair efficiency.	[31,46]

**Table 5:** Nanocarrier testing, validation, and in vivo assessments.

Testing and Evaluation Method	Key Features and Technical Data	Ref.
Physicochemical Characterization (Size, Surface Charge, Structure, Stability)	DLS, TEM, SEM, FTIR, XPS, and zeta potential analysis used for nanoparticle size (~50–200 nm), charge (−30 to +40 mV), and structural confirmation. Ensures colloidal stability (>6 months) and high peptide loading efficiency (>90%).	[2,5,6,9,11,29,32,45,53,54,84,111]
Nanoparticle Functionalization and Conjugation Analysis	Surface Plasmon Resonance (SPR), molecular docking, peptide–protein binding studies confirm efficient functionalization (>80% binding efficiency), stability under physiological conditions, and specificity to target receptors.	[1,18,23,44,59,62,92,94,96,123]
Cellular Uptake and Targeting Efficiency Studies	Confocal microscopy, flow cytometry, and receptor-mediated uptake studies confirm 3–10× higher PF nanoparticle uptake vs. non-functionalized. Uptake via clathrin-mediated endocytosis in cancer cells.	[6,7,41,49,70,77,92,99,102,111]
In Vitro Drug Release and Pharmacokinetics	Controlled release studies (pH/redox-responsive systems) confirm sustained drug release (>90% at pH 5.5 or high-GSH conditions). Drug bioavailability enhanced 3–10× vs. free drugs.	[12,21,53,71,98,100,118]
Cytotoxicity and Biocompatibility Analysis	MTT, Live/Dead, and hemolysis assays confirm high selectivity for cancer cells (>80% viability in normal cells, >90% apoptosis in target cells). Biocompatibility validated in fibroblasts and immune cells.	[5,31,45,50,52,61,67,70,98,103]
In Vivo Tumor Targeting, Biodistribution and Therapy Efficacy	Fluorescence/MRI/PET imaging confirms high tumor uptake (>5% ID/g), improved nanoparticle retention over 24 h, significant tumor growth inhibition (>70%), and extended survival in validated preclinical cancer models.	[2,7,14,17,21,24,40,88,99,112]
Gene Silencing and Genome Editing Efficiency	siRNA and CRISPR studies show >80% gene knockdown efficiency, effective tumor suppression (>60%), and immune system activation (T-cell proliferation and PD-L1 suppression).	[12,15,16,17,21,42,74]
Cancer Imaging and Theranostic (Fluorescence, MRI, PET, CT, SERRS, Photothermal)	Gold nanoparticles (5–15 nm), iron oxide (~10 nm), and quantum dots used for imaging. Tumor detection sensitivity: MRI (75%), PET (80%), and fluorescence (>85%).	[2,23,24,25,26,27,28,44,62,94,112]
BBB Penetration and Neuroprotection	In vivo BBB permeability tests confirm 5–13× enhanced brain uptake, amyloid-beta clearance (70%), and neuronal survival (>80%) in Alzheimer’s and Parkinson’s models.	[4,18,19,42,57,58,95,104,105]
Wound Healing and Regenerative Medicine Testing	Angiogenesis stimulation, collagen deposition (>50% increase), faster wound closure (2×), and infection reduction (>95%) in diabetic models.	[10,30,90]
Antimicrobial and Antiviral Testing	Bacterial eradication (>98%), viral inhibition (100% for SARS-CoV-2 Omicron XBB and RSV), antimicrobial peptide release >30 days.	[10,29,36,37,38,39,116]

**Table 6:** Optimization strategies and future irections in PF nanomedicine.

Category	Key Insights	New Insights and Future Research Directions	Ref.
Nanoparticle Stability and Long-Term Shelf-Life Optimization	Colloidal stability achieved via PEGylation, pH tuning (pH 7.4), and ionic strength control. Storage at −20 °C maintains particle integrity for >12 months. Zeta potential tuning (−20 to +30 mV) prevents aggregation.	Enhancing Stability and Biocompatibility: While PEGylation improves stability, long-term biocompatibility and immunogenic responses should be studied further. The role of secondary stabilizing agents (e.g., polysaccharides and zwitterionic coatings) could be explored. Future research should assess the impact of dynamic biological environments on colloidal stability over time.	[1,21,33,45,52,98]
Enhancing Drug Loading and Encapsulation Efficiency	Lipophilic drugs optimized with lipid nanoparticles (≥90% encapsulation). Hydrophilic drugs benefit from PLGA, chitosan, and silica-based carriers (>85% encapsulation). Cross-linking peptides (RGD and TAT) improve drug retention.	Tailoring Drug Delivery for Personalized Medicine: While high encapsulation efficiency is achieved, future research should focus on optimizing nanoparticle degradation kinetics in different biological environments. Developing patient-specific drug formulations based on genetic or tumor microenvironment profiling could further enhance treatment efficacy.	[5,6,45,53,77,100,118]
Overcoming BBB Limitations	Functionalization with cell-penetrating peptides (TAT and penetratin) and transferrin-receptor-targeting ligands increased CNS drug delivery efficiency by 5–13×. Liposome and polymeric nanoparticles showed the highest BBB permeability.	Expanding to Neurodegenerative and Neuropsychiatric Disorders: While effective for increasing BBB permeability, long-term safety concerns regarding immune responses, off-target effects, and protein corona formation should be addressed. Future research could explore combinations with receptor-mediated transcytosis for even more efficient CNS-targeting.	[4,18,42,57,58,95,104,105]
Reducing Off-Target Toxicity and Improving Tumor Selectivity	Dual-ligand targeting (folic acid + peptides; HA + RGD) improves tumor selectivity by 3–5×. pH/redox-responsive nanoparticles enhance selective drug release (>90% at pH 5.5 or tumor microenvironment).	Refining Tumor-Selective Nanomedicine: While selectivity is improved, integrating AI-driven ligand selection models could enhance precision targeting. Additionally, testing in 3D tumor spheroid models or patient-derived organoids could improve translational predictability.	[9,14,59,69,71,84,98]
Maximizing Cellular Uptake and Internalization Efficiency	Nanoparticle surface charge tuning (+10 to +25 mV) enhances endocytosis. Hydrophobic coatings (lipid-based) increase membrane fusion and intracellular uptake (2–10× compared to non-functionalized carriers).	Optimizing Uptake Pathways for Different Cell Types: Future research could explore how different endocytic pathways (e.g., clathrin-mediated, caveolae-mediated, and macropinocytosis) affect nanoparticle uptake efficiency in specific cancer subtypes. Additionally, the real-time tracking of nanoparticle internalization via super-resolution microscopy could refine targeting strategies.	[7,49,70,92,99,102,111]
Nanoparticle Shape and Morphology Impact on Functionality	Rod-shaped nanoparticles (gold nanorods and mesoporous silica) exhibit superior tumor penetration vs. spherical nanoparticles (up to 4× increased diffusion in tumor matrix). Disordered porous silicon allows for better sustained release.	Shape-Dependent Targeting Strategies: Future work should map out the biological interactions of various nanoparticle shapes in different tumor microenvironments to optimize uptake. Hybrid nanoarchitectures (e.g., combining rods with porous structures) may further enhance functionality.	[1,32,44,51,76,94]
Synergistic Multimodal Therapies for Cancer Treatment	Combination chemo-photothermal, chemo-photodynamic, and gene-silencing therapies demonstrate up to 10× increased tumor inhibition vs. single treatments. Gold nanostars, quantum dots, and hybrid peptide–lipid systems excel in theranostics.	Optimizing Multimodal Approaches for Clinical Translation: While multimodal therapies show promise, challenges include controlling drug release kinetics, mitigating phototoxicity, and optimizing nanoparticle biodistribution. Future work should explore adaptive nanoplatforms that respond to multiple tumor microenvironment cues (pH, hypoxia, enzymes, and ROS levels).	[9,12,27,77,83,99,112]
Scaling-Up and Manufacturing Challenges	Batch-to-batch variation minimized using microfluidic-assisted synthesis and automated peptide conjugation (≤10% variation). Nanoparticle reproducibility controlled via solvent evaporation and controlled pH adjustments.	Improving Industrial-Scale Nanoparticle Manufacturing: While microfluidic-assisted synthesis enhances reproducibility, optimizing continuous flow synthesis methods and real-time quality control monitoring (e.g., AI-assisted process analytics) could further improve scalability and cost-effectiveness.	[3,23,33,47,109]
Enhancing Tumor Imaging and Theranostic Applications	Gold, iron oxide, and quantum dot nanoparticles provide 75–100% detection sensitivity for tumors (MRI, PET, and fluorescence imaging). Functionalization with antibodies and peptides increases specificity.	Next-Generation Multi-Modal Imaging Strategies: While current nanoprobes enhance tumor detection, future efforts should focus on developing biodegradable imaging agents, enhancing multiplex imaging for deeper tissue penetration, and combining diagnostic and therapeutic functions for real-time treatment monitoring.	[2,24,25,26,27,28,44,94,112]
Addressing Antimicrobial Resistance (AMR) and Infection Therapy	PF nanoparticles show >98% bacterial eradication and effective biofilm penetration. Long-term antimicrobial activity achieved with sustained peptide release (>30 days).	Next-Generation Biofilm-Targeting Strategies: While current nanoparticles effectively penetrate biofilms, integrating stimuli-responsive (pH-, enzyme-, or light-sensitive) nanoparticles could enhance targeted bacterial eradication while reducing off-target effects.	[10,29,36,38,39,116]
Advancements in Autoimmune and Inflammatory Disease Treatments	Targeted nanocarriers for arthritis and inflammatory skin conditions.	Joint-homing peptide (ART-1)-functionalized liposomes enhance subcutaneous delivery and preferential accumulation in arthritic joints, leading to improved suppression of arthritis progression. CPP-modified liquid crystalline nanodispersions (e.g., TAT, D4) enhance topical delivery and skin retention of anti-inflammatory agents, reduce cytokine levels (TNF-α, IL-1β), and mitigate oxidative stress in inflammatory skin models.	[33,55]
Emerging Applications in Neurodegenerative Diseases	Alzheimer’s-targeted nanoparticles reduce amyloid-beta aggregation by 60–70%. Parkinson’s-targeted siRNA carriers demonstrate > 80% gene knockdown and neuroprotection.	Optimizing BBB Penetration and Stability: While peptide-functionalized nanocarriers show promise for AD and PD, improving long-term stability and reducing immune response remain key challenges. Future research should explore adaptive nanoplatforms with controlled drug release and responsive delivery triggered by neuroinflammatory signals to enhance specificity and efficacy.	[4,18,19,42,57,58,95,105]
Future Potential: Smart and Responsive Nanomedicine	Real-time biosensing via caspase-3-activated nanoparticles enables cancer therapy monitoring in vivo. Theranostic nanoparticles combining diagnostics and therapy show strong potential for next-generation medicine.	Advancing Personalized Theranostics: The development of biosensing nanoparticles for real-time cancer monitoring is a major breakthrough. However, integrating AI-driven diagnostics with nanoparticle-based sensors could further improve treatment precision. Future studies should explore multiplexed biomarker detection in circulating tumor cells and biodegradable sensor platforms to reduce long-term toxicity.	[94,96,121,122]
